# A recipe for death: Singlet oxygen and type II metacaspase mediate programmed cell death in Chlamydomonas

**DOI:** 10.1093/plphys/kiad661

**Published:** 2023-12-13

**Authors:** Moona Rahikainen

**Affiliations:** Assistant Features Editor, Plant Physiology, American Society of Plant Biologists; Organismal and Evolutionary Biology Research Programme, Faculty of Biological and Environmental Sciences, University of Helsinki, FI-00014 Helsinki, Finland

Programmed cell death (PCD) is a developmental or defensive process that is well characterized in plants and animals. During PCD, a single cell or group of cells undergoes irreversible and tightly regulated self-destruction to allow the rest of the organism to survive. In animals, PCD is executed by caspases that degrade their target proteins promoting the dismantling of the cell (Minina et al. 2017). In plants, evolutionarily ancestral metacaspases play a similar role (Minina et al. 2017). In addition, nitric oxide and reactive oxygen species (ROS) have been shown to be key mediators of stress-induced PCD in plants (op den Camp et al. 2003; Lin et al. 2012). PCD is less studied in unicellular organisms where its benefits from an evolutionary perspective are more complex (Durand et al. 2016). *Chlamydomonas reinhardtii* (hereafter Chlamydomonas) is a unicellular model chlorophyte that forms multicellular structures of several thousand cells and presents social behavior (de Carpentier et al. 2022). Thus, it offers a possibility to study the genetics of social interactions in unicellular photosynthetic organisms (de Carpentier et al. 2022). Interestingly, Chlamydomonas exhibits stress-induced PCD that activates adaptive responses in neighboring cells (Moharikar et al. 2006).

In this issue of *Plant Physiology*, Lambert et al. (2023) shed light on the mechanisms governing PCD in Chlamydomonas. The authors induced PCD in Chlamydomonas using S-nitrosoglutathione (GSNO), which forms a physiological nitric oxide reservoir in Chlamydomonas and is a major trans-nitrosylation agent in protein S-nitrosylation (Morisse et al. 2014). Externally applied GSNO causes nitrosative stress that triggers cell death in Chlamydomonas with characteristics of PCD. Further quantitative proteomic analysis revealed that nitrosative stress downregulates proteins in chlorophyll biosynthesis (Lambert et al. 2023). These changes could result in accumulation of protoporphyrin IX and protochlorophyllide, metabolites that are potent producers of singlet oxygen in light and have been shown to contribute to the ROS-mediated cell death in *Arabidopsis thaliana* (op den Camp et al. 2003; Tarahi Tabrizi et al. 2016). Taking advantage of fluorescent ROS probes, the authors show that GSNO indeed induces the production of singlet oxygen in a light-dependent manner in Chlamydomonas (Lambert et al. 2023). In contrast, GSNO did not trigger PCD in darkness, confirming that the GSNO-triggered PCD is mediated by singlet oxygen in light.

Among the proteins upregulated in response to GSNO, the authors identified a type II metacaspase (MCA-II). Like type I metacaspase (MCA-I), MCA-II is encoded by a single gene in the Chlamydomonas genome. Using reverse and forward genetics, they investigated the role of MCA-I and MCA-II in the execution of PCD. Analysis of Chlamydomonas mutants deficient in either functional *MCA-I* or *MCA-II* revealed that the *mca-II* mutant resists PCD induced either by GSNO or rose bengal, which also generates singlet oxygen in cells under light (Lambert et al. 2023). Moreover, overexpression of *MCA-II-mVenus* under the strong *pPsaD* (PI reaction center subunit II) promoter demonstrated that highly expressed MCA-II promotes cell death in nonstressed Chlamydomonas cultures. Similar phenotypes were not observed for *mca-I* knockout mutant or *MCA-I-mVenus* overexpressor line, confirming that MCA-II is the major metacaspase contributing to the GSNO-triggered PCD in Chlamydomonas (Lambert et al. 2023).

Altogether, the experiments by Lambert et al. (2023) suggest a chain of events where nitric oxide–induced proteomic changes promote the light-dependent production of singlet oxygen that triggers PCD, where MCA-II is the major downstream executor (Fig. 1), although the identification of the mechanisms that cause the observed proteomic changes requires further research. The authors hypothesize that GSNO-induced S-nitrosylation may regulate the degradation of enzymes involved in chlorophyll biosynthesis (Morisse et al. 2014; Lambert et al. 2023). However, it seems likely that these proteins are regulated at multiple levels, which enables the necessary tight control over irreversible PCD. For example, in addition to the putative post-translational regulation, magnesium chelatase subunits working in the chlorophyll biosynthesis have been shown to be transcriptionally regulated in response to nitrosative stress (Kuo and Lee 2021). Similarly, MCA-II activity could be regulated by nitrosylation to allow its timely activation in response to stress signals (Lambert et al. 2023), but another question for further research is to identify the downstream targets of MCA-II in Chlamydomonas.

**Figure 1.**
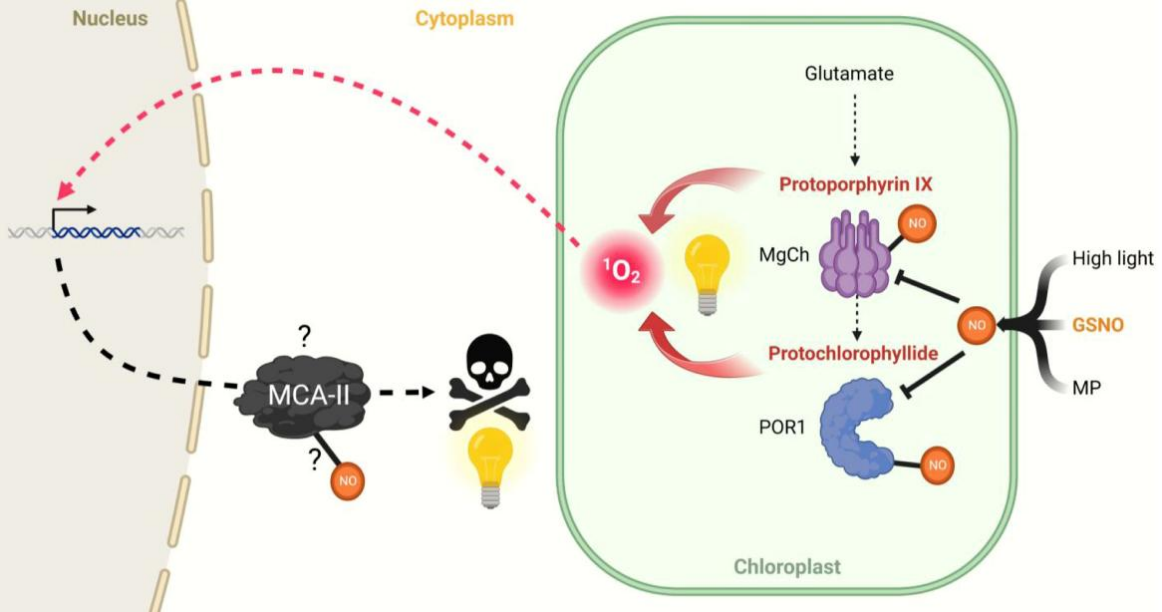
Model of the activation of PCD in Chlamydomonas. Nitrosative stress caused by GSNO downregulates magnesium chelatase (MgCh) and protochlorophyllide reductase 1 (POR1) in chlorophyll biosynthesis. This results in accumulation of protoporphyrin IX and protochlorophyllide and the concomitant production of singlet oxygen (^1^O_2_) in light. Singlet oxygen activates the PCD that is mediated by type MCA-II. Similar nitric oxide–dependent signaling could be activated also by mastoparan (MP) or high light stress. Figure from Lambert et al. (2023).

Research by Lambert et al. (2023) shows that Chlamydomonas undergoes PCD that bears similarities to the PCD observed in vascular plants, suggesting that these mechanisms may have evolved early in the photosynthetic lineage. Moreover, the authors demonstrate that the culture media where cells have undergone heat shock–induced PCD enhances the tolerance of Chlamydomonas cultures toward GSNO, suggesting that molecules secreted from the dying cells can protect neighboring cells from nitrosative stress (Lambert et al. 2023). Similar chemical communication has been observed previously in UV-C–stressed Chlamydomonas cultures (Moharikar et al. 2006). Evidently, PCD of individual Chlamydomonas cells can benefit the whole population, justifying why unicellular organisms display a stress-induced PCD similar to multicellular organisms. However, the putative protective molecules secreted to the culture media remain to be identified. In the future, it will be of interest to study if these molecules are stress specific and if they confer cross-tolerance toward other stressors.

## Data Availability

No new data were generated or analysed in this article.

## Dive Curated Terms

The following phenotypic, genotypic, and functional terms are of significance to the work described in this paper:

POR1 Gramene: Cre01.g015350
POR1 Araport: Cre01.g015350
